# Global emergence and evolution of *Staphylococcus aureus* clonal complex 59

**DOI:** 10.1128/msystems.01492-25

**Published:** 2025-12-31

**Authors:** Shuo Jiang, Peng Gao, Ping Shen, Suying Hou, Chenlu Xiao, Richard Yi Tsun Kao, Pak-Leung Ho, Yonghong Xiao, Huiluo Cao

**Affiliations:** 1 Department of Microbiology, University of Hong Kong, Hong Kong, China; 2 Applied Oral Sciences & Community Dental Care, Faculty of Dentistry, University of Hong Kong, Hong Kong, China; 3 State Key Laboratory for Diagnosis and Treatment of Infectious Diseases, National Clinical Research Center for Infectious Diseases, Collaborative Innovation Center for Diagnosis and Treatment of Infectious Diseases, The First Affiliated Hospital, Zhejiang University School of Medicine 26441, Hangzhou, China; 4 Centre for Virology, Vaccinology and Therapeutics, Hong Kong Science and Technology Park, Shatin, Hong Kong, China; 5 Department of Laboratory Medicine and Clinical Microbiology, Ruijin Hospital Affiliated to Shanghai Jiao Tong University School of Medicine, Shanghai, China; 6 Department of Microbiology, Queen Mary Hospital, Hong Kong, China; Politecnico di Torino, Turin, Italy

**Keywords:** *Staphylococcus aureus*, clonal complex 59, evolution, host adaptation, bloodstream infection

## Abstract

*Staphylococcus aureus* clonal complex 59 (CC59) has emerged as a significant public health threat in Asia, yet the mechanisms driving its host adaptation and global evolutionary success remain poorly understood. Here, we performed a comprehensive genomic analysis of 3,994 global CC59 isolates, which included 549 isolates associated with bloodstream infections from China. Our analysis revealed three phylogenetically distinct lineages exhibiting region-specific distribution patterns, tracing their origins to the USA, Australia, and China. Notably, high-risk CC59 clones circulating in Taiwan likely diverged from mainland Chinese strains during the 1940s-1960s, coinciding with historical population migration following the Chinese civil war around 1949. Among China-associated CC59 strains, respiratory tract colonization was related to high cross-source linkage across multiple ecological niches, suggesting its role as a dissemination hub, particularly for bloodstream infection (BSI). Additionally, we observed significant enrichment of Clf-Sdr family proteins in human isolates, especially in BSI cases. Functional characterization using Δ*clfB* and Δ*sdrD* knockout strains demonstrated impaired biofilm formation, recapitulating findings in USA300. These findings establish an evolutionary framework for CC59 surveillance and highlight promising potential targets for anti-virulence therapeutics.

**IMPORTANCE:**

The prevalence and propagation of *Staphylococcus aureus* clonal complex 59 (CC59) in Asia are serious public health concerns. To understand its adaptation to hosts and worldwide evolutionary success, we analyzed the genomic population structure of all CC59 isolates and traced their evolutionary history. Our research indicates that CC59 lineages developed through unique evolutionary routes that vary across time and space, highlighting their adaptation to diverse ecological environments. This study presents a comprehensive genomic epidemiology framework that integrates extensive metadata analysis with evolutionary assessment. It serves as a model for future *S. aureus* monitoring and provides insights into potential targets for interventions focused on reducing virulence.

## INTRODUCTION

*Staphylococcus aureus* is a Gram-positive opportunistic pathogen causing diverse infections ranging from superficial wounds to life-threatening pneumonia and bacteremia (1). Of particular concern, *S. aureus* bacteremia carries a mortality rate of 20%–30%, posing a significant global health burden (2). The emergence of methicillin-resistant *S. aureus* (MRSA), through acquisition of the staphylococcal cassette chromosome *mec* (SCC*mec*), has exacerbated this threat, establishing MRSA as a leading cause of both hospital-acquired and community-associated infections worldwide (3).

The global distribution of *S. aureus* sequence types (STs) exhibits striking geographical variation. While ST8 (USA300) dominates in the USA, ST239 and ST59 prevail in East Asia (4, 5). Within the Asia-Pacific region, clonal complex 59 (CC59), encompassing ST59 and its single-locus variants, has emerged as particularly prevalent (6). Although current studies primarily focus on regional analyses, particularly in the Asia-Pacific region, CC59 has been sporadically detected in Europe (7), the USA (as USA1000) (8), and Australia (9). Even though significant variability in virulence and antibiotic resistance has been observed in *S. aureus* CC59 lineages between North America and Taiwan (10), the global population structure of CC59 remains poorly characterized. While multiple studies have investigated the local evolution and proliferation of CC59 within Taiwan (11) and mainland China (12), conflicting theories about its origin and dissemination, proposing either a North America origin with subsequent spread to Asia (13), or an initial emergence in Taiwan before global dissemination (10), underscore persistent uncertainties about its evolutionary trajectory.

Bloodstream infections (BSIs) caused by *S. aureus* represent a significant health concern due to their high mortality risk. In 2017, the USA reported approximately 119,247 cases of *S. aureus* BSI, with 19,832 deaths (14). Similarly, a multicenter study in China found *S. aureus* accounting for 7.3% of 2861 BSI cases (15). While MRSA USA300 has been extensively used to study BSIs underlying virulent mechanisms including *sarZ* mutation-mediated regulation of clumping factor B (ClfB) proteins to enhance its virulence (16), the pathogenesis of the Asian-dominant CC59 strain is rarely reported. ClfB belongs to microbial surface components recognizing adhesive matrix molecules (MSCRAMMs), which mediated staphylococcal adherence to host extracellular matrix components. A recent study using USA300 revealed that clumping factor-serine aspartate repeat protein (Clf-Sdr) mutation enhanced biofilm formation (17). Comparative studies of ST59 and ST239 from BSIs show ST59 exhibits greater enrichment of staphylokinase (*sak*) and chemotaxis inhibitory protein (*chp*) genes (5, 18). Furthermore, ST398-t571 isolates demonstrate a distinct hemolysin profile from ST59-t437 strains, suggesting the gamma-hemolysin component B (*hlgb*) gene may confer a selective advantage in human blood (19). Current research has primarily focused on identifying regional high-risk BSI clones and comparing CC59 with other clonal complexes. However, the intra-lineage evolution of CC59 leading to enhanced virulence in BSIs, along with its specific pathogenic mechanisms, remains largely unexplored.

To address these knowledge gaps, we performed a comprehensive genomic analysis of global *S. aureus* CC59 strains, integrating our collections with public databases. Our study aims to elucidate population lineage variations, characterize resistance and virulence gene profiles, identify key pathogenesis determinants, and reconstruct evolutionary trajectories through time-scaled phylogenetic analysis.

## MATERIALS AND METHODS

### Genome collection, sequencing, assembly, and quality control

Our data set comprises a diverse subset of *S. aureus* CC59 genomic sequences, sourced from both our internal collection and publicly accessible database. In our collection, a total of 551 *S*. *aureus* CC59 isolates were gathered between 2011 and 2024 from 41 hospitals located across 22 provinces in China. Each strain was identified to the species level using MALDI-TOF MS spectrometry. The sequencing of these 551 isolates was carried out on the Illumina HiSeq X-Ten platform, utilizing a 2 × 150 bp paired-end read configuration. Quality control of the raw sequencing data was conducted using FastQC v0.23.2 (20) with default settings. Subsequently, high-quality reads were assembled using SPAdes v3.15.5 (21) with the default setting. In addition, we downloaded 3,180 assembled genomes from the GenBank database and 58 assembled genomes from PubMLST, as of the last access on 15 December 2024. Moreover, a total of 205 short-read data sets available in the NCBI SRA were assembled using SPAdes v3.15.5, culminating in a data set of 3,994 *S*. *aureus* genomes for subsequent analysis. All assembled genomes were confirmed as CC59 based on a scheme allowing only one allelic difference from ST59 by mlst v2.22.1 (https://github.com/tseemann/mlst). All genomes were confirmed as *S. aureus* using GTDB-Tk v2.4.0 (22). The specific schemes applied for CC59 identification are listed in Table S1. To ensure the quality of the genomes, CheckM v1.1.6 (23) was utilized to assess both the completeness and contamination of each genome. Only genomes with a completeness of over 95% and contamination of less than 5% were retained for further analysis.

### Genomic annotation and population structure

All genomes were annotated using Prokka v1.14.5 with default setting (24). The Comprehensive Antibiotic Resistance Database (CARD v4.0.0) was utilized to detect antibiotic resistance genes (ARGs) using the Resistance Gene Identifier (RGI v6.0.3) with strict parameters (25). Virulence factors (VFs) were identified by performing a BLASTp search against a curated database (Table S2) with 60% coverage and identity. *Spa* typing of all *S. aureus* isolates was conducted using spaTyper v0.2.1 (https://github.com/mjsull/spa_typing). SCC*mec* typing was performed using Bactopia v3.0.0 with the StaphopiaSCCmec module (26). Prophage annotation was carried out using an in-house script (https://github.com/JasonJiang42/S.aureus_CC59_analysis/blob/main/PHASTEST_API.sh) to automate PHASTEST API submission (27). Intact prophages were extracted and annotated with Pharokka v1.7.5 (28). Annotated genomes in GFF format were used as input for Roary v3.13.0, and the pangenome was identified using default thresholds and settings (29). The pairwise Jaccard similarity coefficient was calculated using the pangenome matrix, retaining those with an index over 0.5 using the *pw_similarity.py* script from GraPPLE (30). All assemblies were mapped to the *S. aureus* M013 reference genome (NCBI reference: NC_016928.2) to generate core genome single nucleotide polymorphisms (SNPs) alignments using Snippy v4.6.0 (https://github.com/tseemann/snippy). FastBAPS v1.1.4 was employed to identify lineages of genetically similar isolates within the SNPs alignment (31, 32).

### Phylogenetic analysis

Phylogenomic analysis was performed to call core-genome SNPs (cgSNPs) from genome alignment generated by Snippy v4.6.0 (33). The recombination sites were removed with Gubbins v2.4.1 (34). A maximum-likelihood phylogenetic tree was then constructed using IQ-TREE v2.2.03 (35) based on core genome SNP alignments after recombination removal.

### Bacterial genome-wide association studies

GWAS analysis was conducted by comparing human host vs other hosts, as well as human blood vs other human sources within the collection (Fig. S1a). To identify genes putatively associated with the potential of virulence in *S. aureus* CC59, we used the presence and absence matrix of VFs from prior identification as input to Scoary v1.6.13 (36). Detected associations were reported for all genes passing a significance threshold of <0.05 following the Benjamini-Hochberg correction. The various lengths of k-mer were generated by fsm-lite v1.0 using the default setting (https://github.com/nvalimak/fsm-lite). Pyseer v1.3.10 was used for a GWAS based on *k-mer* composition across all collections. *K-mers* significantly associated with distinct traits were further mapped to the *S. aureus* M013 reference genome to identify putative trait-specific genes (37).

### Gene deletion

To carry out gene deletion in the chromosome for *clfB* and *sdrD*, we utilized pKOR1 and primers listed in Table S3. The insertion fragment was generated through crossover PCR, involving two rounds. The first-round PCR amplified a 1-kb fragment upstream with primer P1 and P2 and downstream of the gene stop codon with primer P3 and P4. The second-round PCR combined the 1 kb upstream fragment and 1 kb downstream fragment into a single amplified product with primer P1 and P4. This 2-kb fragment was then ligated into a linearized pKOR1 plasmid using cold fusion (Vazyme), and the plasmid’s accuracy was confirmed via sequencing. Once transformed into RN4220 and aim strains through electro-transformation, the replacement of the gene was selected following the pKOR1 protocol (38). The deletion of the target gene without flanking regions in the chromosome was also verified through sequencing.

### Phenotypical validation

Deleted *clfB* and serine-aspartate repeat-containing protein D (*sdrD*) genes were generated for two CC59 isolates, D4396 and D4275, following a previously described method (39). Both wild-type and gene-inactivated *S. aureus* strains were grown in tryptic soy broth until reaching an optical density at 600 nm (OD_600_) of 1. The cultures were then diluted to an OD_600_ of 0.01 and incubated at 37°C with shaking at 200 rpm for 24 h. OD_600_ was taken every 30 min to create growth curves. These growth curves were subsequently analyzed using a permutation test, executed by the *compareGrowthCurves*() function in statmod version 1.50 (40), with 50,000 permutations specified. To assess biofilm formation, *S. aureus* cultures were initially cultivated in TSB at 37°C for 18 h to reach the OD_600_ to 0.01. Following this, 200 μL of the culture was transferred into 96-well plates and incubated for an additional 24 h at 37°C. After incubation, the wells were washed three times with PBS, and 25 μL of a 1% crystal violet solution was added to each well. The plates were then incubated at 37°C for 15 min. Post-incubation, the crystal violet was removed, and the wells were again washed three times with PBS. Subsequently, 200 μL of 30% acetic acid was added to each well, and the plates were incubated at room temperature for 10 min. Finally, the absorbance was measured at 600 nm using a Multiskan SkyHigh Photometer (Thermo Fisher Scientific, Massachusetts, USA).

### Cross-source linkage of Chinese strains

To investigate *S. aureus* CC59 in China, we selected 2,045 genomes isolated from this region. SNP alignment for all genomes was performed as described previously. The pairwise SNP distance matrix was generated using SNP-dists v0.8.2 (https://github.com/tseemann/snp-dists) from the core genome alignment without recombination removal. Previous studies have established genetic relatedness cutoffs for *S. aureus*, indicating that a threshold of 12 cgSNPs can detect 95% of transmission events within a similar timeframe (41). As we lack supporting epidemiological data, we defined cross-source linkage in China as instances where pairwise genomes exhibited fewer than 12 cgSNPs and were isolated in the same year (42). For genome pairs carrying less than 12 cgSNPs, we extracted these SNPs and mapped them to specific gene location within the reference genome (GenBank accession NC_016928.2).

### Bayesian inference of *S. aureus* CC59

From the data set, we selected 2,720 genomes with well-documented collection times and locations for phylogeographical analysis. The temporal origins of *S. aureus* CC59 were estimated using a maximum-likelihood tree analyzed with BactDating v1.1.1 (43), employing a mixed clock model. Phylodynamic inference of the effective population size and time-scaled phylogenies was conducted using skygrowth v0.3.1 (44). Geographic transmission patterns were estimated using TreeTime v0.11.4 with the migration model (45).

### Statistical analysis and visualization

The enrichment analysis of VFs and ARGs within each BAPS lineage was performed using Fisher’s exact test with Benjamini-Hochberg correction for multiple tests. Group comparisons were made using the Wilcoxon signed-rank test. All networks in this study were created using Cytoscape v3.9.1 (46). Genetic alignments were visualized with pyGenomeViz v1.5.0 (47), and phylogenetic trees were plotted using iTOL v6 (48). Other visualizations were produced using ggplot2 v3.4.4 (49).

## RESULTS

### China as the predominant geographic source of *S. aureus* CC59

**Fig 1:**
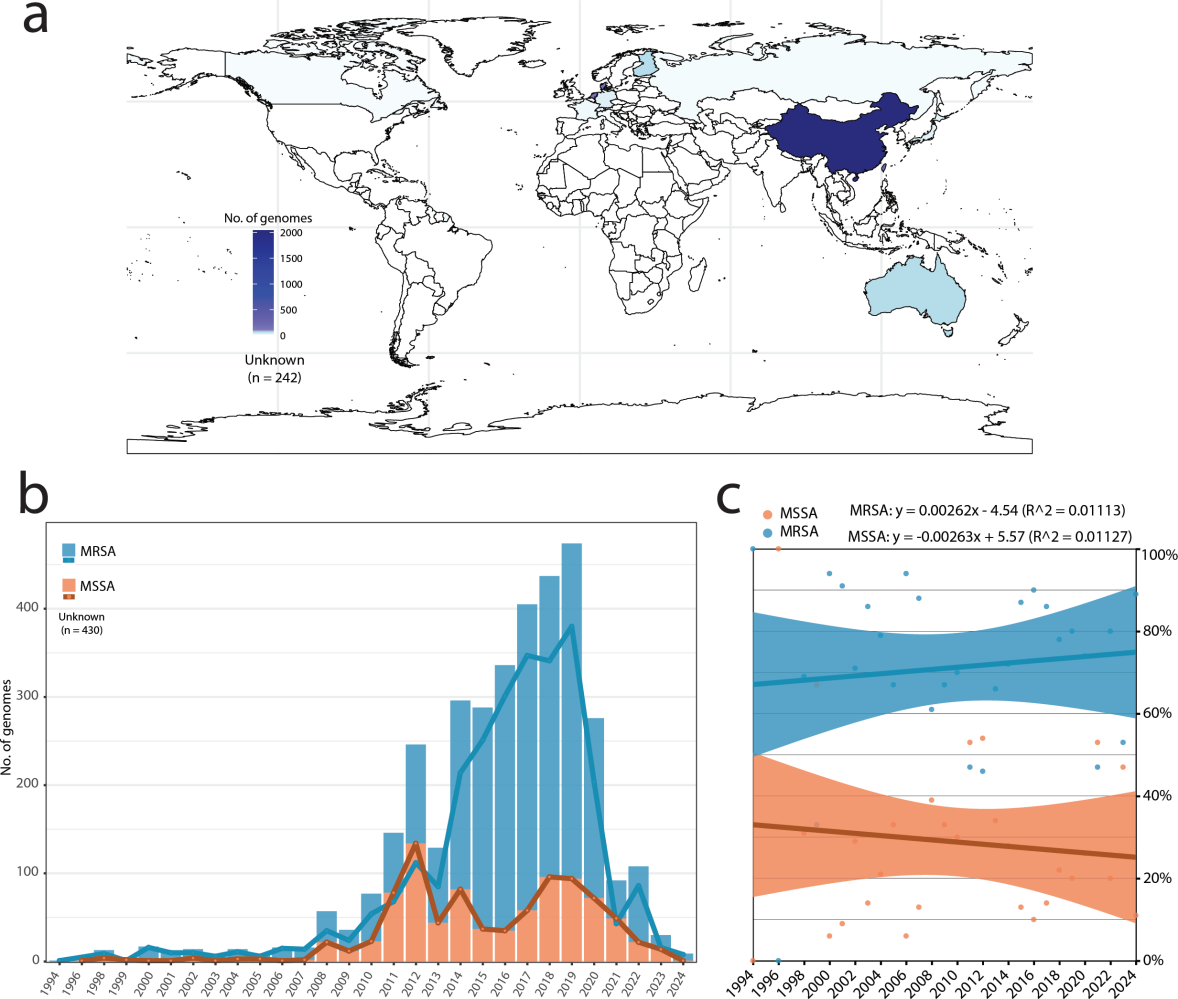
Global distribution of collected *S. aureus* CC59. (**a**) Geographical distribution illustrating the number of *S. aureus* CC59 genomes included in the analysis, with 242 genomes lacking detailed regional information. The map file was downloaded from ChiPlot v1 (https://www.chiplot.online/). (**b**) Temporal timeline depicting the number of MRSA and MSSA genomes collected between 1994 and 2024, noting that 430 genomes lacked detailed regional information. (**c**) Scatter plots showing the proportion of MRSA and MSSA over time, with both trends regressed using linear expressions.

A total of 3,994 *S*. *aureus* CC59 genomes were analyzed, comprising 551 clinical isolates from 41 Chinese hospitals and 3,443 genomes sourced from public databases (Table S4). Of these, 242 lacked detailed geographical information. The remaining 3,752 genomes spanned 29 geographical regions, with mainland China exhibiting the highest prevalence (51.2%, 2,045/3,994) (Fig. 1a). Among Chinese isolates, 32 provincial-level areas were represented though 827 lacked specific provincial attribution (Fig. S1b). Zhejiang accounted for the highest proportion (6.60%, 135/2,045), followed closely by Hubei (6.3%, 129/2,045). The isolates were collected between 1994 and 2024, with a peak in 2018–2019 (Fig. 1b). MRSA represented 71.9% (2,873/3,994) of the collection, with a marginal increase over time, as indicated by a linear regression model (Fig. 1c). Chinese isolates contributed disproportionately to the MRSA burden (91.1%, 1,863/2,045) compared to non-Chinese regions (55.2%, 943/1,707; *P* < 0.01, *χ*² test) (Fig. S1c). The majority of isolates (86.8%, 3,466/3,994) originated from human hosts, with bloodstream infections, representing the predominant clinical source (29.2%, 1,013/3,994), including 666 from China (Table S4).

### Genomic population structure reveals three distinct *S. aureus* CC59 lineages

**Fig 2:**
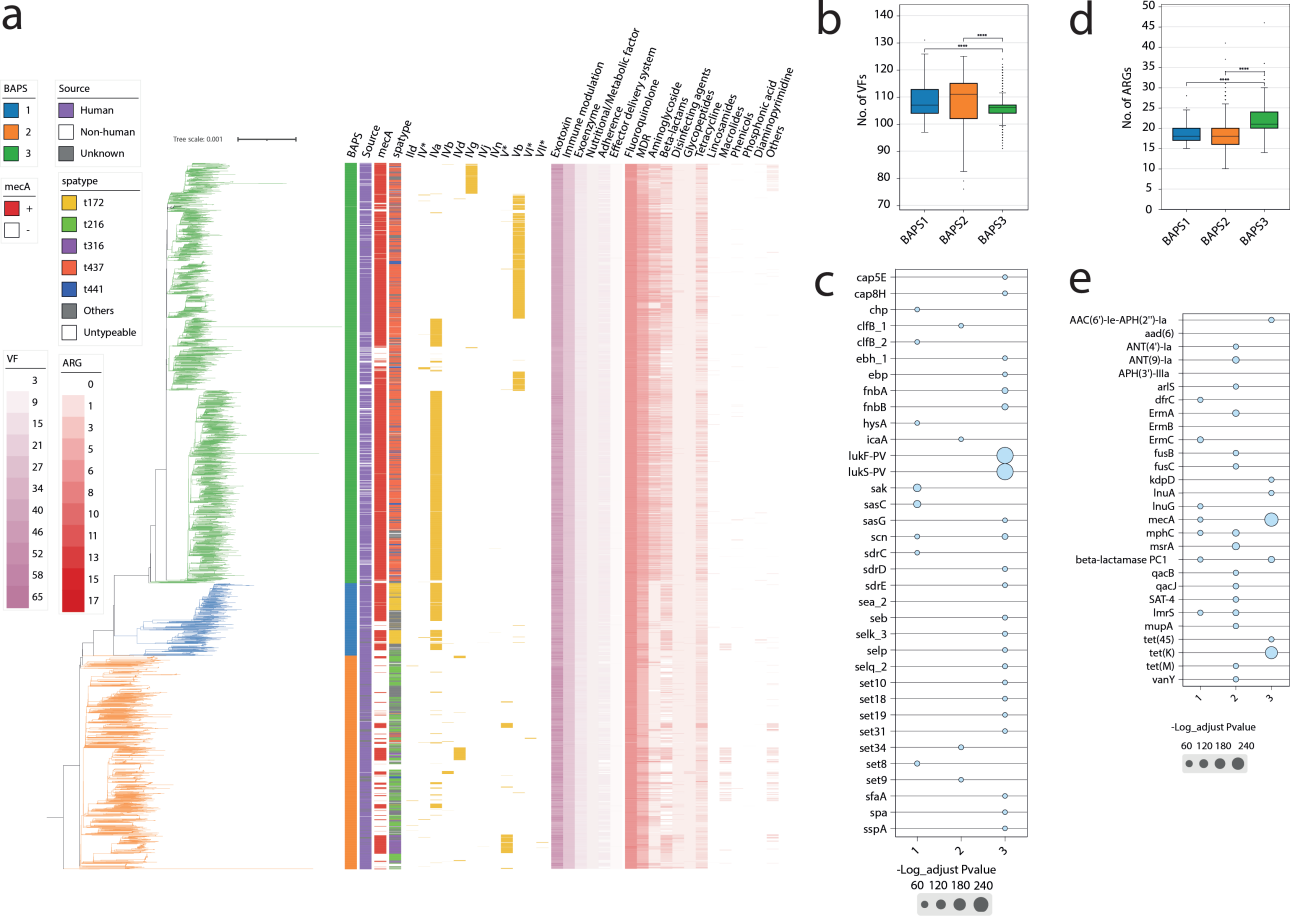
Phylogenetic analysis of 3,994 *S*. *aureus* CC59 genomes. (**a**) A mid-rooted, maximum-likelihood phylogenetic tree of 3,994 *S*. *aureus* genomes, accompanied by metadata including BAPS clusters, source, *mecA* presence, *spa* type, and a heatmap illustrating VFs and ARGs. Branches are color-coded according to the BAPS clusters. The tree was plotted using iTOL v6 (48). (**b**) and (**d**) Comparison of ARG and VF counts per genome across BAPS clusters, respectively. Statistical analysis was performed using the Wilcoxon test (two-sided). In the boxplots, the box represents the interquartile range (IQR), encompassing 50% of the central data, with the central line denoting the median. (**c**) and (**e**) Enrichment analysis of ARGs and VFs across BAPS clusters, respectively. The *x*-axis represents the BAPS clusters, while the *y*-axis lists the genes. The size of the bubbles indicates the negative logarithm of the adjusted *P*-value, calculated using Fisher’s exact test.

A maximum-likelihood phylogeny was reconstructed using 63,833 recombination-free SNPs derived from 3,994 genomes, with M013 as the reference genome (Fig. 2a). Following recombination removal, a total of 1,863 sites were eliminated using the Gubbins process (Fig. S2a). The majority of these sites were located within the SCC*mec* region and prophage regions (Fig. S2b). The average genome size was 2.8 Mbp, yielding a pangenome of 17,356 genes and a core genome of 1,311 genes (Table S5). Population structure analysis revealed three genetically distinct clusters (BAPS1-3) (Fig. 2a), all predominantly composed of ST59. BAPS1 (410 genomes), BAPS2 (1,208 genomes), and BAPS3 (2,376 genomes) exhibited intra-cluster median SNP distances of 157, 115, and 75, respectively, compared to an inter-cluster median of 198 ± 24.9 (*P* < 0.01, Wilcoxon’s test) (Fig. S3). Secondary prevalent STs included ST375 (18.8%, 77/410) in BAPS1, ST87 (25.1%, 303/1,208) in BAPS2, and ST338 (9.6%, 228/2,376) in BAPS3 (Table S4). BAPS3 is predominantly a Chinese lineage, comprising 75.9% (1,803/2,376) of the genomes, while *S. aureus* CC59 in BAPS2 is widely distributed in the USA (38.7%, 468/1,208) and the UK (28.8%, 348/1,208). The BAPS1 lineage is globally distributed, with approximately half originating from China (59.0%, 242/410), followed by the European region (22.4%, 92/410) (Fig. S4).

Among 118 identified *spa* types, t437 predominated globally (45.7%), with cluster-specific distributions: t172 in BAPS1 (55.1%, 226/410), t216 in BAPS2 (49.6%, 599/1,208), and t437 in BAPS3 (75.5%, 1,793/2,376) (Fig. 2a; Table S4). MRSA dominated the BAPS1 and BAPS3 clusters, representing over 70% of each group, while MSSA accounted for 70.9% of the BAPS2 cluster. The *mecA* gene was primarily located on the SCC*mec* type IVa (40.8%, 1,630/3,994), followed by SCC*mec* type Vb (19.1%, 761/3,994). Notably, genomes carrying SCC*mec* type Vb were mainly from the BAPS3 cluster, accounting for 99.5% (757/761) of these genomes, predominantly originating from China (Fig. 2a; Table S4).

A total of 163 VF genes were identified in *S. aureus* CC59, primarily associated with exotoxin production, immune modulation, exoenzyme activity, nutritional/metabolic factors, adherence mechanisms, and effector delivery systems (Fig. 2a; Table S6). Notably, the number of VFs related to exotoxin and immune modulation was higher compared to other categories (*P* < 0.01, Wilcoxon’s test). BAPS3 carried fewer VFs compared to the other clusters, with a median of 106 ± 4.8 (*P* < 0.01, Wilcoxon’s test) (Fig. 2b). However, it is noteworthy that the two-component toxin *lukS-PV*/*lukF-PV* was significantly enriched in BAPS3, along with *set* genes related to exotoxin production (Fig. 2c). The ARG profile included 79 genes, with all genomes carrying genes conferring resistance to fluoroquinolones (*nor* and *arl* gene clusters) (Fig. 2a; Table S7). Further comparison across the BAPS clusters revealed that BAPS3 carried a higher number of ARGs, with a median of 21 ± 3.5 (*P* < 0.01, Wilcoxon’s test) (Fig. 2d). Additionally, ARGs conferring resistance to beta-lactam antibiotics and tetracycline were notably more prevalent in genomes from BAPS1 and BAPS3 clusters (Fig. 2d). Specifically, the enrichment of ARGs in BAPS3 included the presence of *mecA* and *tet(K)* (Fig. 2e). In contrast, BAPS2 exhibited a high number of enriched ARGs, such as *ant*, *qac*, and *fus*.

### Prophage diversity within *S. aureus* CC59

**Fig 3:**
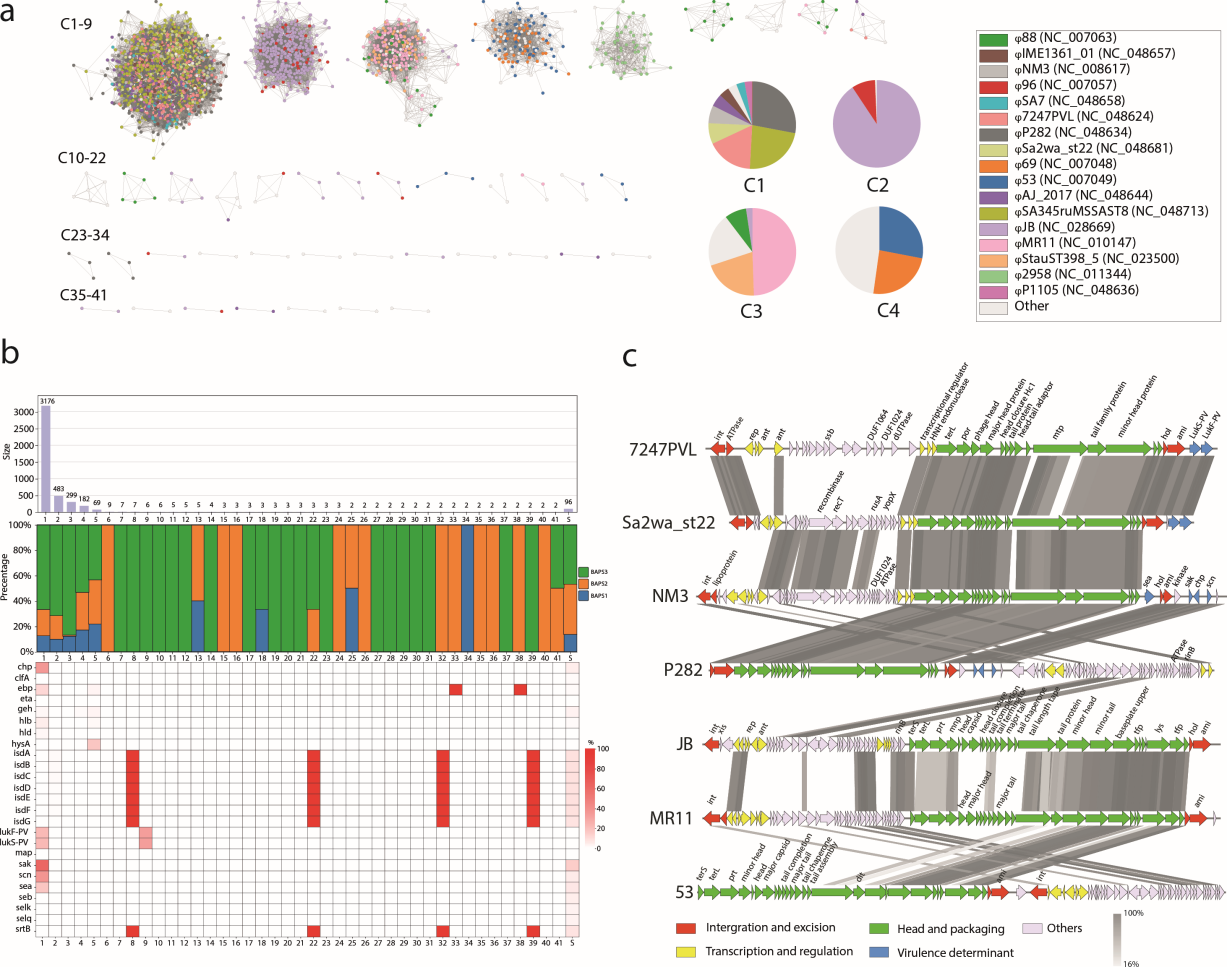
Genetic characteristics of prophages from *S. aureus* CC59. (**a**) Pairwise similarity network of 4,423 putative prophages based on shared genes. Each node represents a prophage, with colors indicating reference prophages. The embedded pie chart presents the distribution of prophages among clusters C1 to C4. The network was created using Cytoscape v3.9.1 (46). (**b**) Comparison of prophages across clusters. The bar chart displays the number of prophages in each cluster, while the stacked bar plot illustrates the proportion of prophages from the three BAPS clusters. The heatmap below shows the proportion of VFs carried by prophages in each cluster. (**c**) Genetic alignment of seven representative prophages. The annotation of coding sequences is indicated above the arrows, with arrow colors representing phage functional categories. Genetic alignments were visualized with pyGenomeViz v1.5.0 (47).

As one of the highly recombinant regions in *S. aureus* CC59, all intact prophages were extracted, resulting in the identification of 4,423 prophages. The pangenome matrix categorized these putative prophages into 41 clusters and 96 singletons, utilizing a pairwise Jaccard similarity coefficient threshold of >0.5. Aside from clusters C1 through C5, other clusters contained fewer than 10 prophages each (Fig. 3a). The largest cluster, C1, comprised 3,176 prophages and exhibited the most variability, comprising 17 different prophages. The predominant prophage was φP282, accounting for 28.0% (889/3,176), followed by φSA345ruMSSAST8 at 22.9% (727/3,176) (Fig. 3b). Clusters C2 and C5 primarily consisted of φJB and φ2958PVL, representing 90.7% (438/483) and 75.4% (52/69) of their respective prophages. In terms of BAPS distribution, clusters C1–C5 were present in genomes from all three BAPS clusters. However, phage cluster C3 was predominantly found in BAPS1 and BAPS3, with only a small number present in BAPS2 (1.0%, 3/299) (Fig. 3b). The majority of prophage clusters carried a limited number of VFs, ranging from 0 to 8, with a mean of 1.6 ± 1.5 (Table S8). Cluster C1 contained a significantly higher number of VFs compared to all other groups (*P* < 0.01, Kruskal-Wallis test) (Fig. S5), with exotoxins (*lukS-PV/lukF-PV* and *sea*), exoenzymes (*sak*), and immune modulation (*scn*) enriched (Fig. 3b). Specifically, φ7247PVL and φSa2wa_st22 carried *lukS-PV/lukF-PV*, while φNM3 carried an immune evasion cluster comprising *sea*, *sak*, *chp*, and *scn*. Genetic alignment of seven representative prophages in *S. aureus* CC59 revealed the basic structure of the integration region, head and packaging region, and regulatory regions (Fig. 3c).

### Clf-Sdr proteins drive host adaptation in *S. aureus* CC59

**Fig 4:**
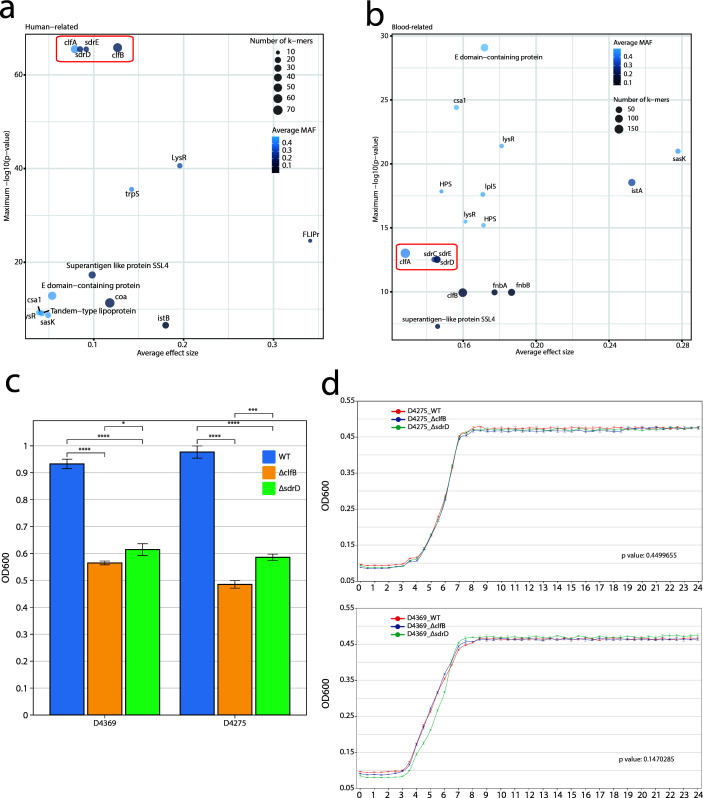
Bacterial genome-wide association studies and phenotypic assays. Significance levels and average effect sizes of genes with *k-mers* identified as significant in a bGWAS involving human-derived isolates (**a**) and bloodstream isolates (**b**), with a red box highlighting genes associated with the MSCRAMM family. (**c**) Biofilm formation ability of wild-type (WT) and mutant strains D4369 and D4275. Statistical analysis was conducted using the Wilcoxon signed-rank test. (**d**) Growth curves for WT and mutant strains D4369 and D4275. Statistical analysis was conducted using permutation tests by the *compareGrowthCurves*() function (40).

To identify the genetic determinants associated with human infection, a bGWAS was performed, comparing 3,466 human-derived isolates with 353 non-human-derived isolates. The VF matrix-based bGWAS identified 15 VFs that were significantly different between the two groups (Table S9). Notably, clumping factor B (*clfB*), which mediates *S. aureus* clumping in human blood plasma, was significantly enriched in human isolates, demonstrating over 60% sensitivity and specificity. The bGWAS also identified 3,712 k-mers associated with human isolates, corresponding to 141 genes, including *sdrD*, *sdrE*, *clfA*, and *clfB*, which belong to the MSCRAMM family (Fig. 4a; Table S10). An extensive comparative analysis was performed focusing on bloodstream infections, which included 1,013 genomes from human blood samples and 1,221 isolates from other human sources. Utilizing a VF matrix-based bGWAS approach, 16 VFs were found to be significantly more prevalent in bloodstream isolates. Notably, MSCRAMM such as *sdrD*, *sdrE*, *sdrC*, and *clfB* were detected with high sensitivity, exceeding 80% (Table S11). Additionally, a k-mer-based bGWAS revealed 2,039 k-mers associated with bloodstream genomes, linked to 54 genes (Table S12). Consistent with pangenome analysis, the *sdr* and *clf* genes were significantly enriched in bloodstream isolates (Fig. 4b).

To investigate the involvement of MSCRAMM genes in the invasion process of *S. aureus* CC59, knockout mutants for the *clfB* and *sdrD* genes were generated using strains D4275 and D4369 from BAPS1. Biofilm formation assays demonstrated a notable reduction in biofilm formation in the BAPS1 knockout strains (Wilcoxon test, *P* < 0.01) (Fig. 4c). Knocking out *clfB* or *sdrD* gene did not significantly alter the growth rates of the BAPS1 strains (*P* > 0.1) (Fig. 4d).

### Cross-source linkage and SCC*mec* replacement in *S. aureus* CC59 from China

**Fig 5:**
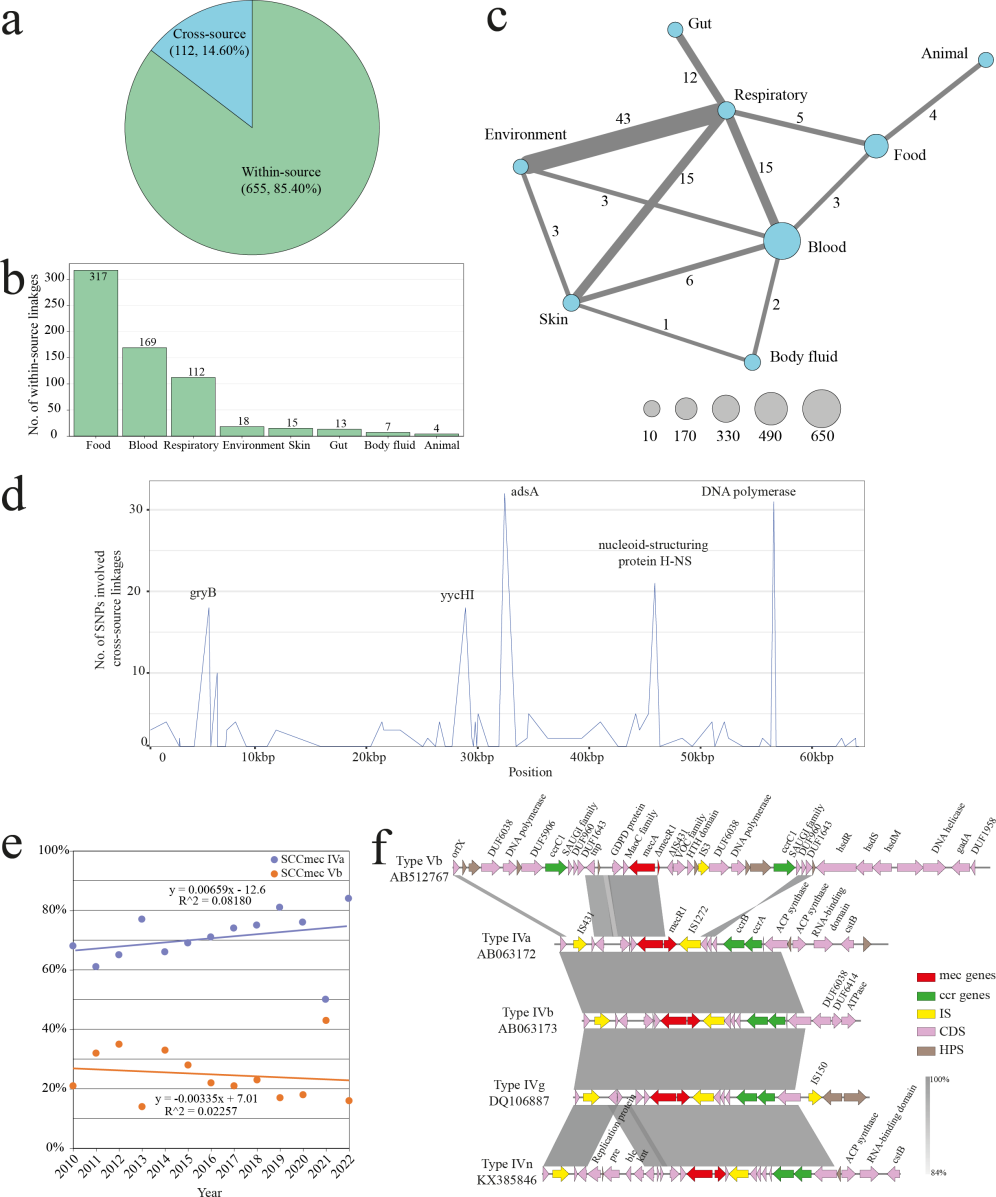
Cross-source linkages and SCC*mec* among *S. aureus* CC59 in China. (**a**) Proportion of within- and cross-source linkages. (**b**) The bar plot indicates the number of within-source linkages in each source group. (**c**) The network diagram illustrates the number of cross-source linkages, with edge weights representing the number of linkages and numbers labeled. The size of each node reflects the number of genomes involved in cross-source linkages for each source. The network was created using Cytoscape v3.9.1 (46). (**d**) The line plot displays the number of SNPs associated with genomes involved in the cross-source linkages, with the *X*-axis representing the chromosome location of the reference genome M013 (GenBank accession: NC_016928.2). (**e**) The scatter plot depicts the proportion of SCC*mec* IVa and Vb genomes in strains from China over time. (**f**) Genetic alignment of SCC*mec* structures, with annotations of coding sequences, indicated above the arrows. Arrow colors represent functional categories. Genetic alignments were visualized with pyGenomeViz v1.5.0 (47).

To further investigate the China-endemic CC59 strains, 2,045 strains from China were analyzed with 32.6% (666/2,045) from BSI (Table S4). The prevalence of Clf-Sdr family proteins was significantly higher in Chinese isolates compared to those from other countries, with BSI isolates exhibiting a particularly elevated mean of 4.8 ± 0.6 (Fig. S6). To further explore cross-source linkage, a SNP-based method was employed to identify genome pairs from the same year with fewer than 12 SNP differences, resulting in 767 genome pairs (Fig. 5a). The majority of these pairs (85.4%, 655/767) occurred within the same source group, predominantly originated from food sources (48.4%, 317/655), followed by blood (25.8%, 169/655) and respiratory samples (17.1%, 112/655) (Fig. 5b).

Among genomes involved in cross-source linkage, a significant proportion of putative interactions was observed between respiratory sources and other five sources, including human gut, environment, skin, blood, and food samples (38.4%, 43/112), demonstrating a higher colonization shift ability between them (Fig. 5c). All 268 SNPs to define cross-source linkage were annotated through being mapped to the genome of M013 (NC_016928.2) (Fig. 5d), resulting in 252 sites mapped to coding regions (Table S13). A total of 34 genome pairs were observed to possess SNPs at the *adsA* gene locus, which is responsible for the synthesis of adenosine, an immune evasion factor during infection. SNPs were also identified in genes coding for quinolone resistance, such as GyrB, nucleoid-structuring protein H-NS, and DNA polymerase. Additionally, SNPs were detected in proteins YycH and YycI, which regulate the expression of *S. aureus* autolysins (Fig. 5d). The VF profile of *S. aureus* CC59 in China indicates that genomes isolated from the respiratory tract carry the highest number of accessory VFs (*n* = 30) among clinical isolates. The accessory VFs shared across different sources are mainly associated with capsule synthesis (*cap*) and exotoxin production (*sel*) (Table S14).

Given that the majority of *S. aureus* genomes in China are MRSA (Table S4), the distribution of SCC*mec* types among these MRSA genomes was further analyzed. SCC*mec* type IVa was predominant (66.9%, 1,368/2,045), followed by type Vb (20.5%, 419/2,045) (Fig. S7). Temporal analysis revealed a slight increase in the isolation of SCC*mec* IVa *S. aureus* CC59, as indicated by linear regression, while a decrease in the detection of SCC*mec* Vb genomes was observed from 2010 to 2022 (Fig. 5e). In addition to the dominant SCC*mec* IVa and Vb types, SCC*mec* types IVb, IVg, and IVn were also detected among *S. aureus* CC59 strains in China (Fig. S7). Genetic alignment revealed that these SCC*mec* elements possessed a modular structure comprising two essential components: the *mec* complex, which includes the *mecA* gene and the *mecR1* regulator, and the *ccr* complex, containing *ccr* genes that facilitate the mobility of the cassette. Compared to the IV types, SCC*mec* Vb exhibited a more complex structure with additional genetic content, featuring duplicated *ccr* genes and extra joining regions (Fig. 5f).

### Evolutionary trajectories of *S. aureus* CC59

**Fig 6:**
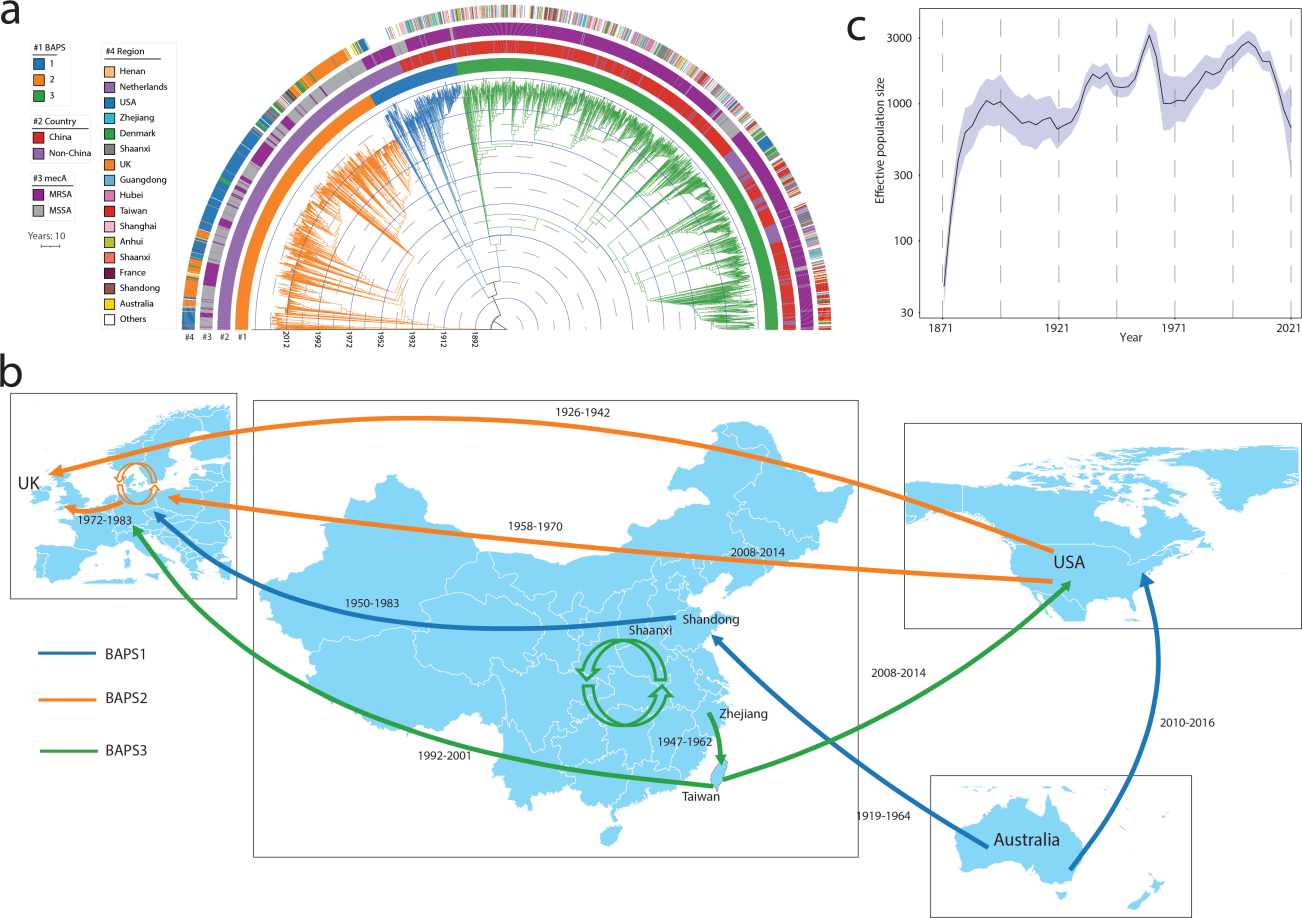
Phylogeography and transmission of *S. aureus* CC59. (**a**) Spatiotemporal phylogenetic tree of 2,720 *S*. *aureus* genomes. Branch colors represent different BAPS lineages. The outer arcs display metadata, including BAPS lineages, country of origin, *mecA* positivity, and regional classification. (**b**) Global transmission routes of *S. aureus* CC59. Numbers adjacent to each line indicate the inferred year of transmission by BactDating v1.1.1 (43). Line colors correspond to the associated BAPS lineages. The map file was downloaded from ChiPlot v1 (https://www.chiplot.online/). (**c**) Dynamics of effective population size of *S. aureus* CC59 over time. The 95% confidence intervals are depicted as shaded areas.

Among all 2,720 *S*. *aureus* CC59 strains with detailed location and date information, the most recent common ancestor of the CC59 strains was estimated to have existed in 1872 (95% CI: 1840–1892), subsequently evolved into three distinct BAPS lineages (Fig. 6a). These lineages exhibit distinct regional specificity. BAPS1, originating in 1896 (95% CI, 1883–1925), is more divergent than BAPS2 and BAPS3 and can be subdivided into two clades: one from China and the other from non-China regions. In contrast, BAPS2, which originated in 1910 (95% CI: 1859–1908), comprises strains exclusively from non-China regions. Meanwhile, BAPS3, which emerged in 1927 (95% CI: 1891–1933), primarily circulated in China (Fig. 6a). As *S. aureus* CC59 strains are found to be geographically widespread, symmetrized migration rates were estimated on a global scale. The USA, UK, mainland China, Taiwan, Australia, and several other European regions, including the Netherlands, Denmark, and France, were associated with high levels of internal CC59 migration (Fig. S8). BAPS1 is estimated to have originated in Australia and was subsequently transmitted to China in 1952 (95% CI: 1919–1964) and to the USA in 2012 (95% CI: 2010–2016). BAPS2, primarily consisting of MSSA, originated in the USA and then migrated to the UK in 1936 (95% CI: 1926–1942). This lineage later spread to the Netherlands in 1965 (95% CI: 1958–1970), leading to its circulation throughout Europe around the 1970s (Fig. 6b). Although BAPS1 was introduced to China in 1952, it did not result in significant circulation within the region, accounting for only 11.8% of all Chinese genomes. In contrast, BAPS3, which constitutes 88.2% of the strains, is the predominant lineage in China. It is estimated to have originated from Shaanxi in 1927 (95% CI: 1981–1933). Subsequently, widespread colonization and migration were observed in provinces such as Anhui, Zhejiang, Hubei, Shanghai, and Shaanxi. Notably, the Taiwan clone within the BAPS3 lineage is estimated to have migrated from mainland China in 1958 (95% CI: 1947–1962) and was later transmitted to Europe in 1997 (95% CI: 1993–2001) and to the USA in 2012 (95% CI: 2008–2014) (Fig. 6b). The effective population size of *S. aureus* CC59 demonstrated an expansion beginning in 1921, reaching its peak around 1956. Following this peak, a slight decrease was observed between 1956 and 1966. After 1966, the population size began to expand again, indicating a renewed period of genomic growth (Fig. 6c).

## DISCUSSION

The evolution and pathogenesis of *S. aureus* CC59 have garnered considerable attention in recent years, largely due to its high prevalence in Asia. While previous studies have primarily focused on regional analyses in Taiwan (11) and mainland China, CC59 has also been consistently reported in the USA (8), Australia (9), and Europe (7), albeit at lower prevalence. To elucidate the global population structure of this clinically important lineage, we conducted an extensive genomic analysis incorporating geographically diverse isolates, which revealed three phylogenetically distinct CC59 lineages with characteristic regional distributions. Our temporal reconstruction indicates CC59 experienced peak clonal expansion in 1956, followed by decline after methicillin introduction in 1959 (50), then subsequent resurgence coinciding with MRSA emergence in 1966 (51). Phylogeographic evidence supports a model of concurrent divergence in North America and East Asia, forming distinct sub-lineages shaped by regional selection pressures (10). Our analysis of over 2000 genomes corroborates this, revealing the divergent evolution of BAPS2 (USA-associated) and BAPS3 (China/Taiwan-associated) lineages, which emerged during the 1910s–1920s. These findings provide a comprehensive spatiotemporal framework for understanding the clonal transmission dynamics and population expansion of CC59.

The BAPS1 lineage, predominantly consisting of *spa*-type t172 strains, exhibits limited global prevalence, which may be linked to its lower pathogenicity, as reflected by a reduced number of VFs. This is further supported by sporadic reports of BAPS1 *spa*-type t172 infections, including asymptomatic carriers in South Finland (2007–2016) (7) and bloodstream infections in Chinese children (2012–2020) (52). Observations in a previous study (9) in Western Australia indicated that one local clone is closely related to the Taiwan clone, whereas other strains were presumed to be USA-related strains. However, the true diversity within the study and routes of transmission remains unrecognized. Here, we traced the BAPS1 lineage to Australia, with subsequent transmission to East Asia (1920s–1960s) and the USA (2010s), resolving prior uncertainties about its relationships to the Taiwan and USA clones.

Notably, most strains in the BAPS2 lineage, also known as USA1000 (CC59-*spa*-t216), exclusively originated outside China, predominantly in the USA (8) and the UK (53). In contrast to the predominant presence of USA300 in the USA, reports of USA1000 are limited in North America (10), and its relationship with Asian strains remains unknown. Although USA1000 strains were MRSA-carrying SCC*mec* IV, the majority of *spa*-type t216 strains in our collection were MSSA, suggesting that USA1000 may have adaptively evolved through the SCC*mec* acquisition in the USA. Additionally, BAPS2 isolates harbor fewer ARGs compared to isolates from BAPS1 and BAPS3. This discrepancy likely reflects regional antibiotic usage patterns, particularly the non-prescription use of antibiotics in Asia (54). Consistent with a previous study comparing the Taiwan ST59 and USA1000 strains (13), there was no evidence to suggest that the Taiwan ST59 strain emerged from the USA. Beyond the USA strains, our research further delineates the distinct lineages of Asian and European strains supported by evidence of differing ARG profiles and geographic niche exclusion.

In contrast to the MSSA-dominant BAPS2 lineage, BAPS3 lineages are primarily composed of MRSA. The BAPS3 lineage primarily comprises *spa*-type t437 strains originating from China, which carry SCC*mec* types IV and V. Previous epidemiological analyses indicate that the community-associated CC59-t317-SCC*mec* IV lineage is predominant in China, with diverse clonal isolates circulating across different provinces (55). The higher number of ARG (*mecA*, *tetK*) in BAPS 3 may provide an advantage for its dissemination in China where community use of antibiotics is common (56). Additionally, CC59 strains with SCC*mec* V in BAPS3, known as the Taiwan clone, have undergone additional evolution by acquiring SCC*mec* VII, a variant related to SCC*mec* V, in Taiwan (57). The presence of various SCC*mec* types, coupled with heightened pathogenic potential evidenced by severe inflammatory reactions and tissue destruction in animals (58), likely contributes to the widespread dissemination and severity of infections associated with the BAPS3 lineage. Evidence of the concurrent and independent evolution of the Taiwan strain and the BAPS1/BAPS2 lineages is well-documented (10, 13). However, the origin of the Taiwan CC59 clones and their relationship with strains in mainland China remains unclear. Our data suggest that these clones likely originated from mainland China between the 1940s and 1960s, providing clarification on their evolutionary history. Although epidemiological data on *S. aureus* in mainland China before 1998 is largely lacking (59), MRSA emerged early in the 1980s in Hong Kong and Japan which provides a clue to the early emergence of *S. aureus* in China around the 1950s. Previous analysis revealed that returning from travel has been documented as leading to potentially detrimental *S. aureus* outbreaks within the community and healthcare settings (60). In alignment with historical developments, the transmission of *S. aureus* CC59 might be associated with the migration of 2 million Chinese to Taiwan following the civil war around 1949. Unlike the internal evolution observed in mainland China, Taiwan plays a significant role in the global dissemination of *S. aureus* CC59. We observed dual evolutionary patterns in Taiwan: one clade spread to Europe in the 1990s, while another reached the USA around the 2000s, aligning with a previous study of 258 Taiwan isolates proposing two clades diverging in the 1980s (11).

The replacement and expansion of ST59 in China, through shifts in SCC*mec* type and acquisition of VFs, are well explained by comparison with ST239 (5). Focus on *S. aureus* CC59, the internal evolutionary changes, and genetic pattern shift in different hosts and BSI has not yet been reported. In addition to SCC*mec*, PVL-positive prophages, and VFs, particularly adhesion elements may serve as crucial drivers of *S. aureus* global spread and host adaptation. Through bGWAS analysis, we estimated that the dominant pathogenesis of CC59 is associated with the MSCRAMM family, which comprises Clf-Sdr and Cna families (61). Our analysis revealed significant enrichment of the Clf-Sdr family in humans, particularly bloodstream isolates. To functionally validate their role in CC59, we performed *in vitro* biofilm assays using *ΔclfB* and *ΔsdrD* knockout strains, which exhibited impaired biofilm formation, consistent with observations in USA300 strains (17). Complementation studies in USA300-ΔMSCRAMMs (Clf-Sdr) mutants demonstrated that restored biofilm formation via fibrinogen binding and intercellular adhesion mechanisms linked to persistent musculoskeletal infections (17). A further study using USA300 in a murine BSI model comprehensively revealed the mechanism by which *clfB* contributes to the hypervirulence of the *sarZ* mutant by altering virulence regulation (16). Another study on *S. aureus* CC8 using the NCTC8325 strain validated the interaction of SdrD with desmoglein 1, which is involved in adhesion to human cells (62). Apart from *S. aureus* CC8, a *clfB* mutant in *S. aureus* CC1 and CC30 exhibited significantly reduced binding to corneocytes from patients with atopic dermatitis (63). Although several CCs have been reported to show altered adhesion due to Clf-Sdr mutations, there is currently no validation on CC59 strains regarding the role of Clf-Sdr in host adaptation. Similar to USA300 hypervirulence, our work provides the first experimental validation of the role of the Clf-Sdr protein in contributing to the Asian-dominant CC59 BAPS1 in human invasive infection. Although the knockout model utilized strains from BAPS1, the genomes in BAPS2 and BAPS3 were derived from CC59, which exhibit similar genomic structures. These results underscore the role of Clf-Sdr adhesins as potential drivers of the global spread, host adaptation, and persistence of *S. aureus* CC59.

Although numerous studies have studied CC59 in China, the driving force to its spread is not clear. We observed a higher number of Clf-Sdr proteins in Chinese BSI isolates, which may confer an enhanced ability for biofilm formation. To further investigate the China-endemic CC59 strains, we examined the genetic relatedness of CC59 across different colonization sites. Based on a previous study estimating a substitution rate of approximately 5 whole-genome SNPs or 3 core-genome SNPs (cgSNPs) per genome per year (41), we applied a cutoff of 13 cgSNPs which would capture 95% of *S. aureus* transmission events within the same timeframe (41). Our analysis revealed that CC59 strains colonizing the respiratory tract were more likely to transmit to other isolation sources, likely due to multiple transmission routes, including direct contact, fomites, droplets, and aerosols (64). The ability of *S. aureus* to colonize tissues such as the epithelium of the respiratory, vascular, and intestinal systems is highly related to MSCRAMMs, the *agr* quorum-sensing system, and the capsule (65). Therefore, the high presence of accessory VFs related to the *cap* and *clf-sdr* genes in respiratory tract isolates could explain their capacity for tissue invasion. The *sak* gene shared by *S. aureus* from the respiratory tract and other colonization sites can enhance the bacterium’s local invasiveness and its ability to penetrate host tissues (66). This capability might be a crucial factor in the *S. aureus* potential for re-colonization. In between-source transmission, we frequently observed mutations in *adsA*, a gene linked to enhanced host adaptation through proline metabolism modulation (67). Additionally, genes involved in DNA replication and antibiotic resistance (e.g., *gyrB*) were associated with between-source transmission. Although our identified SNPs in GyrB have not been reported to increase quinolone resistance currently, the mutations involved in the putative transmission may contribute to the fitness adaptation of *S. aureus*. Prior studies on other bacteria suggest that DNA polymerase mutations can improve adaptive fitness in *E. coli* via negative feedback regulation (68), while the Thr-86-Ile mutation in GyrA enhanced fitness in *Campylobacter* (69). These findings indicate that the fitness changes in *S. aureus* CC59 are crucial for its re-colonization of different human sites, providing genomic evidence for further experimental validation. Furthermore, SCC*mec* types IV and V dominate in China, with reports indicating their gradual replacement of types I, II, and III in hospital settings (70). While larger cassettes, such as types II and III, enhance MRSA survival in hospitals, the smaller type IV cassette may confer an evolutionary advantage by facilitating horizontal gene transfer (71). This could explain the increasing prevalence of type IV over type V in China.

While this study has limitations including potential temporal and spatial biases from using predominantly public genomic data sets with incomplete metadata, and the need for future *in vivo* validation of Clf-Sdr family proteins in CC59 pathogenesis, it makes several key advances. We provide the first demonstration that the global *S. aureus* population is primarily dominated by three distinct clonally expanded lineages, each with unique genetic signatures, and establish Clf-Sdr adhesions as critical virulence determinants in human infections. Our findings reveal that these lineages emerged through spatiotemporally distinct evolutionary pathways, reflecting adaptation to different ecological niches. Collectively, this study establishes an integrated genomic epidemiology framework that combines large-scale metadata analysis with evolutionary characterization, offering both a blueprint for future *S. aureus* surveillance and insights into targets for virulence-focused interventions.

## Data Availability

The genomic data are available from NCBI Bioproject under accession codes: PRJNA1248392 and PRJNA749878. The codes used in this study are available at https://github.com/Ultramancc/S.aureus_CC59_analysis. The genomic data collected in this study are uploaded to figshare (https://figshare.com/articles/dataset/Th_genome_data_used_in_S_aureus_CC59_analysis/30362707?file=58750732).

## SUPPLEMENTAL MATERIAL

Fig. S1 msystems.01492-25-s0001.tif Distribution of MRSA and MSSA genomes across regions and isolated source groups.

Fig. S2 msystems.01492-25-s0002.tif Analysis of recombination removal.

Fig. S3 msystems.01492-25-s0003.tif Comparison of SNP distance across BAPS lineages.

Fig. S4 msystems.01492-25-s0004.tif Regional distribution of CC59 genomes across BAPS lineages.

Fig. S5 msystems.01492-25-s0005.tif Comparison of number of VFs across prophage clusters.

Fig. S6 msystems.01492-25-s0006.tif Number of proteins carried by CC59 isolates from different countries and comparison of Clf-Sdr proteins from Chinese genomes.

Fig. S7 msystems.01492-25-s0007.tif Distribution of types of SCCmec carried by Chinese CC59 strains.

Fig. S8 msystems.01492-25-s0008.tif Transmission migration rates across different region.

Supplemental Tables msystems.01492-25-s0009.xlsx Tables S1-S14.

ASM does not own the copyrights to Supplemental Material that may be linked to, or accessed through, an article. The authors have granted ASM a non-exclusive, world-wide license to publish the Supplemental Material files. Please contact the corresponding author directly for reuse.
