# Supplementary figures and images for: Global emergence and evolution of Staphylococcus aureus clonal complex 59
Source: mSystems. 2025 Dec 31;11(2):e01492-25. doi: 10.1128/msystems.01492-25 (PMC12911391; doi:10.1128/msystems.01492-25)

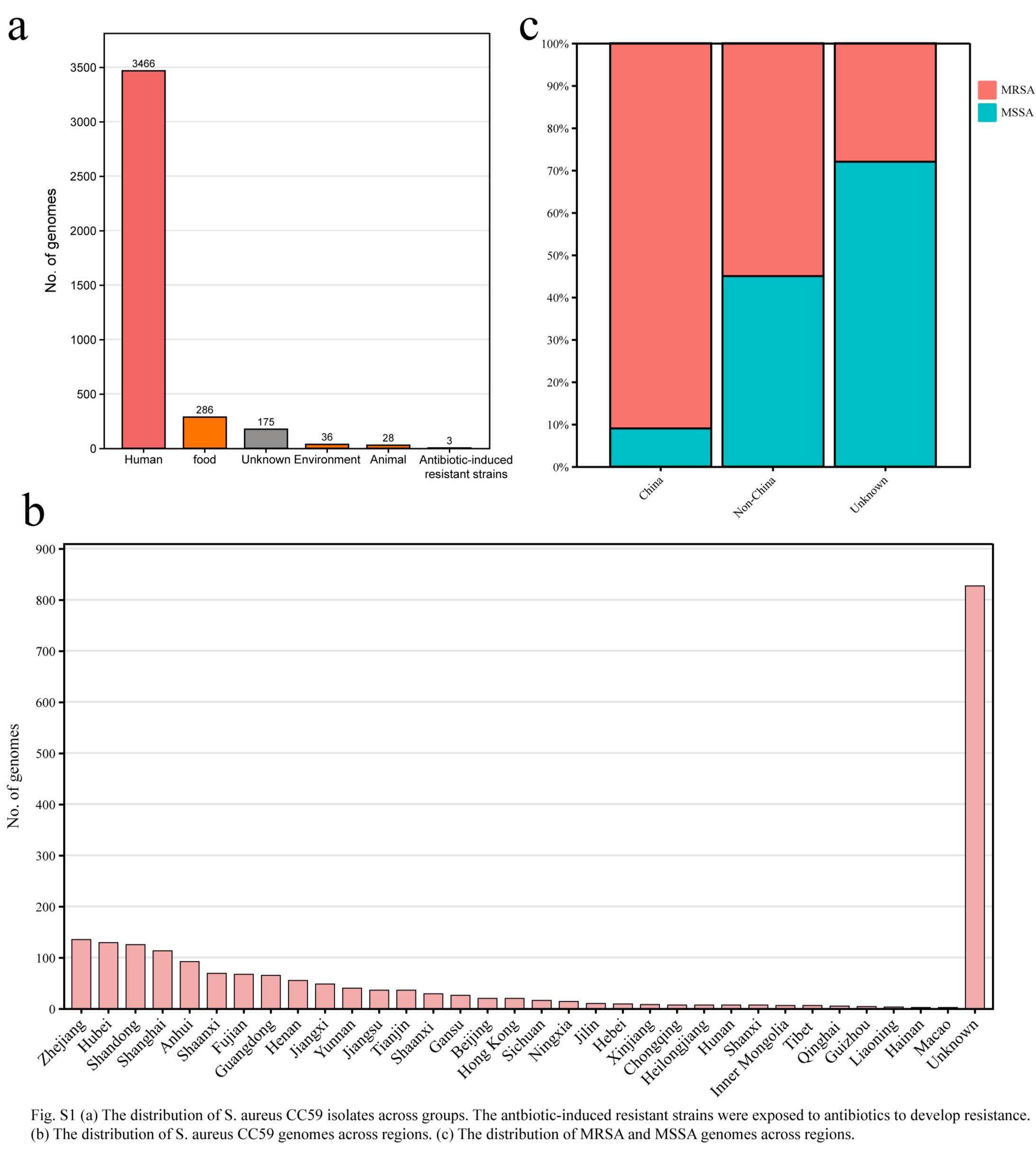

Supplement: Fig. S1 — Distribution of MRSA and MSSA genomes across regions and isolated source groups. [file msystems.01492-25-s0001.tif]

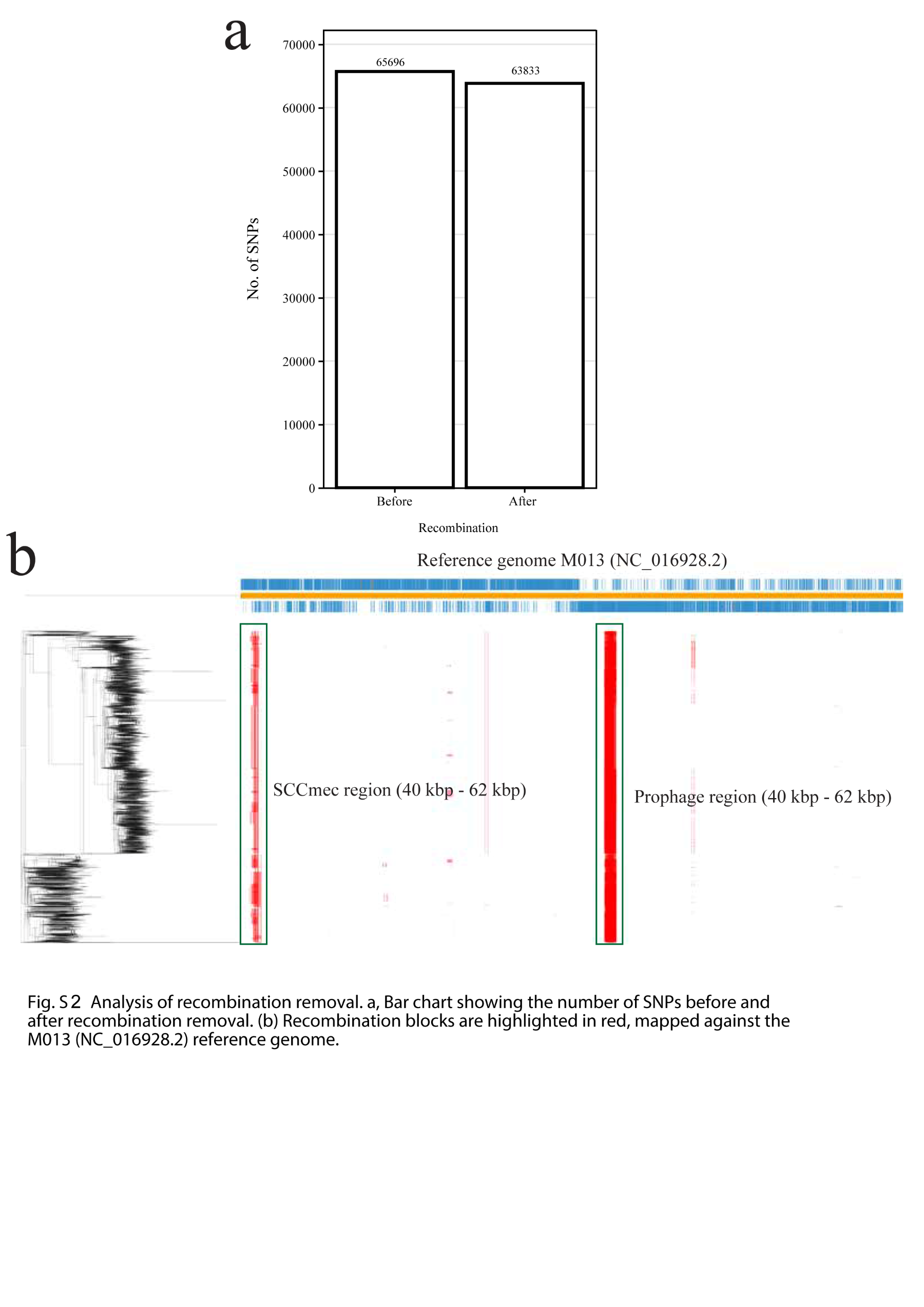

Supplement: Fig. S2 — Analysis of recombination removal. [file msystems.01492-25-s0002.tif]

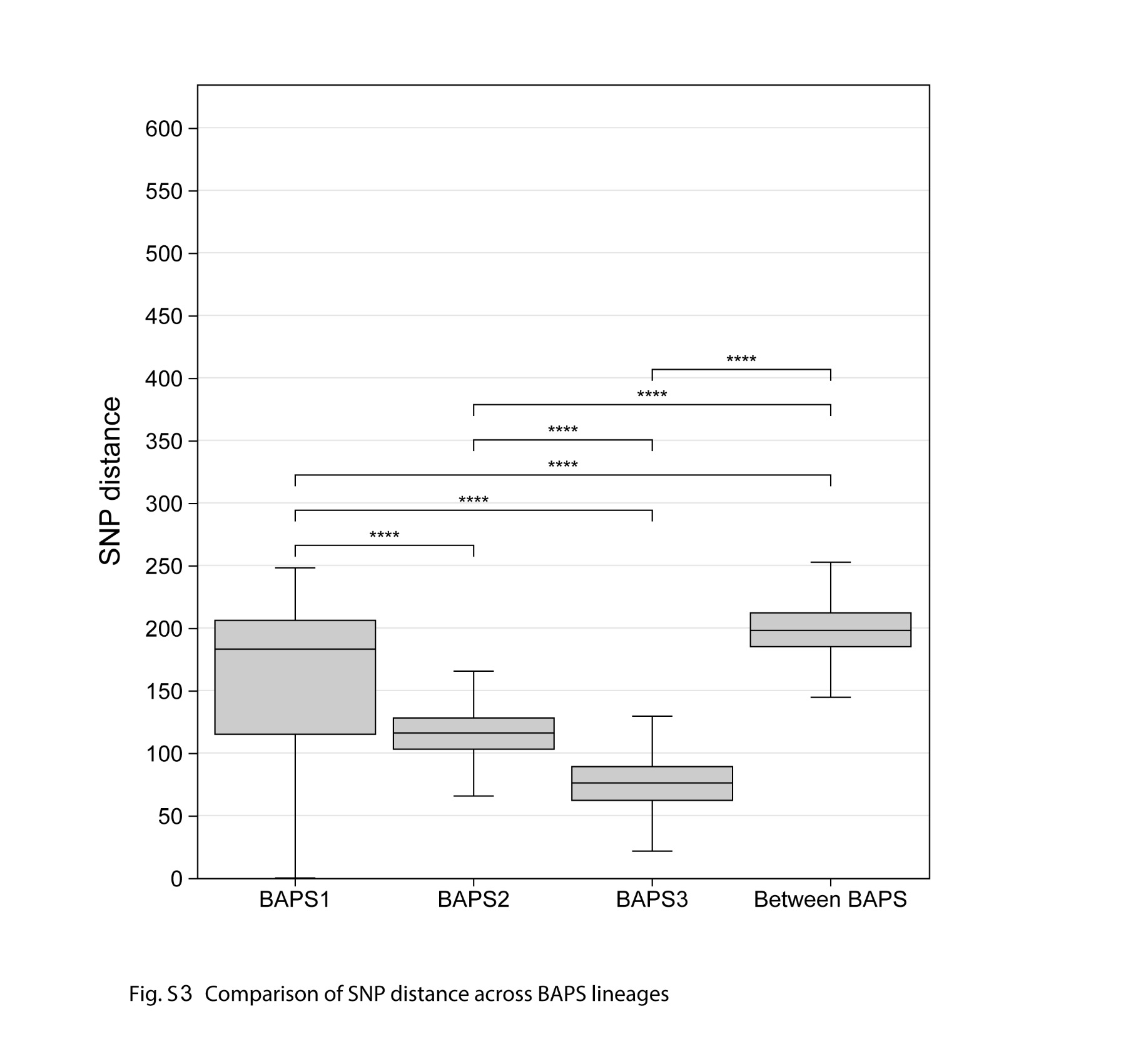

Supplement: Fig. S3 — Comparison of SNP distance across BAPS lineages. [file msystems.01492-25-s0003.tif]

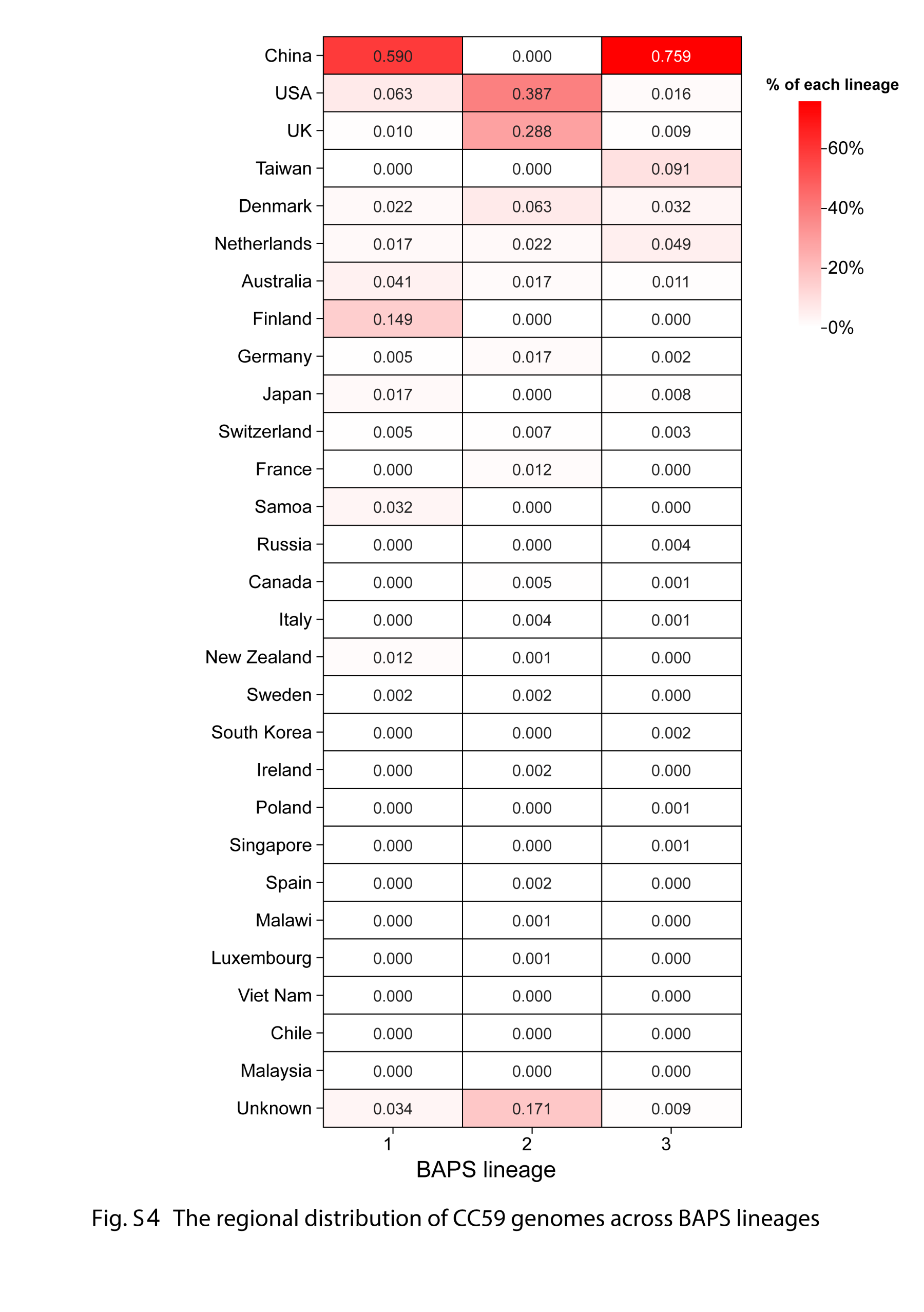

Supplement: Fig. S4 — Regional distribution of CC59 genomes across BAPS lineages. [file msystems.01492-25-s0004.tif]

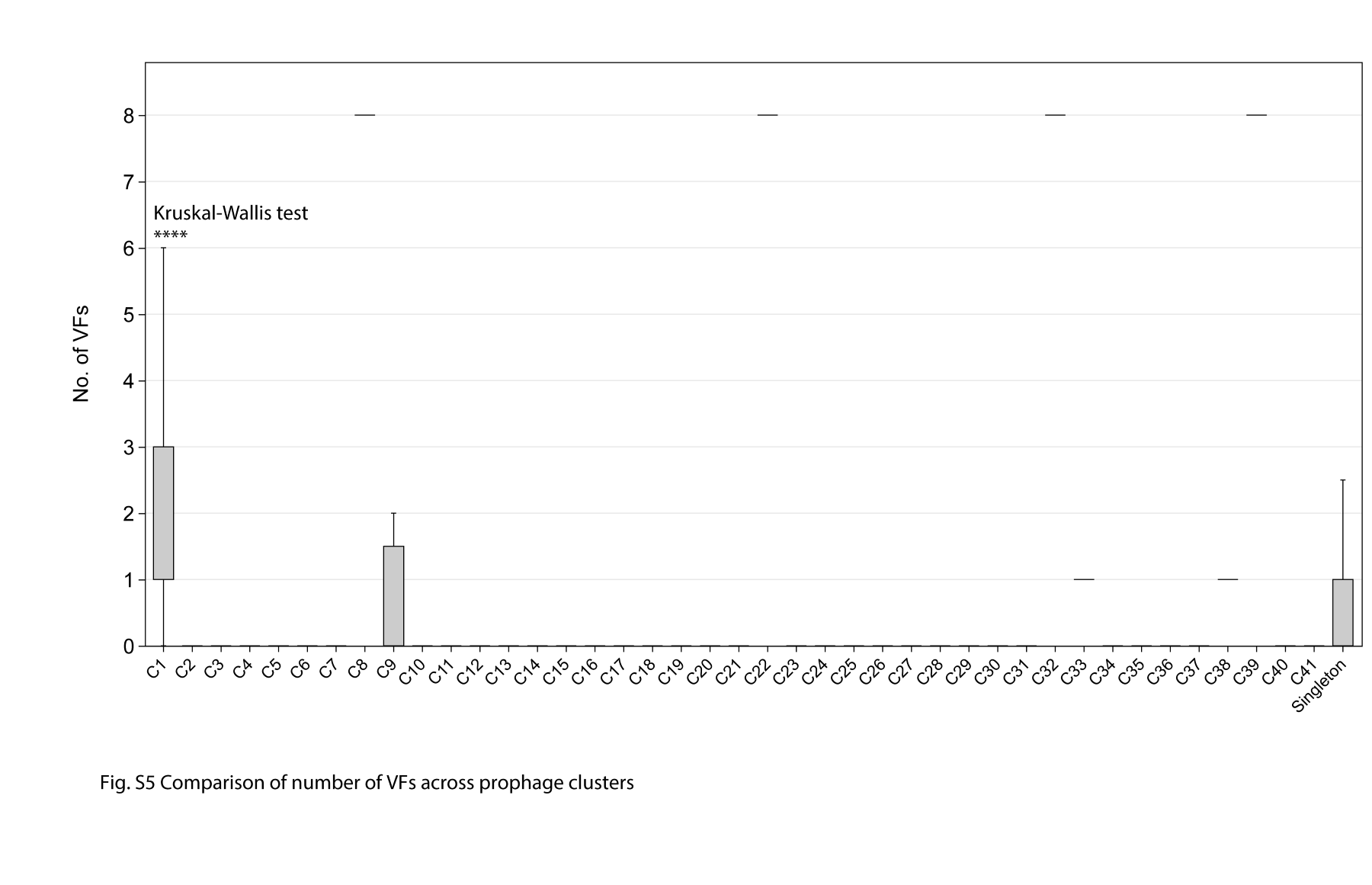

Supplement: Fig. S5 — Comparison of number of VFs across prophage clusters. [file msystems.01492-25-s0005.tif]

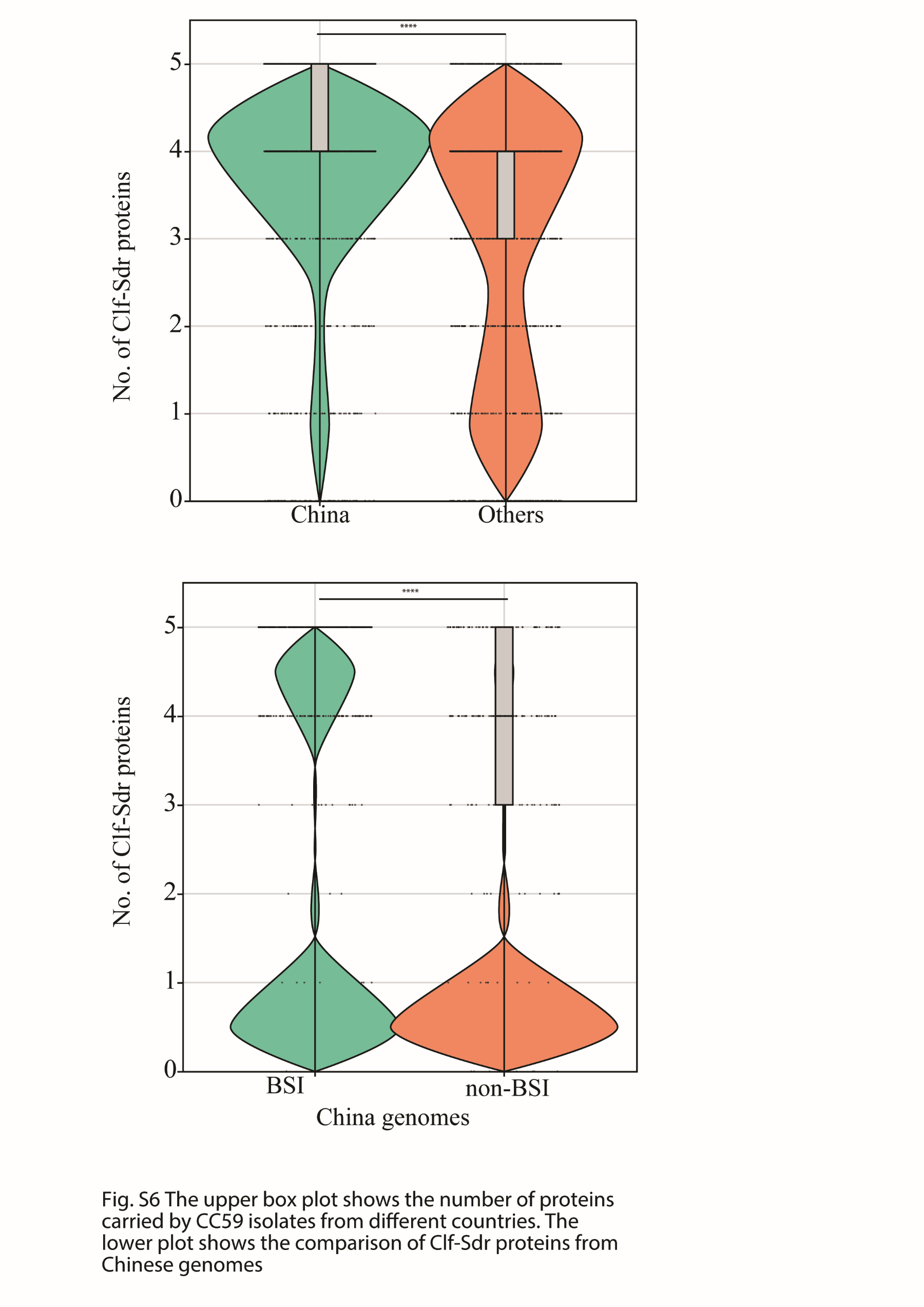

Supplement: Fig. S6 — Number of proteins carried by CC59 isolates from different countries and comparison of Clf-Sdr proteins from Chinese genomes. [file msystems.01492-25-s0006.tif]

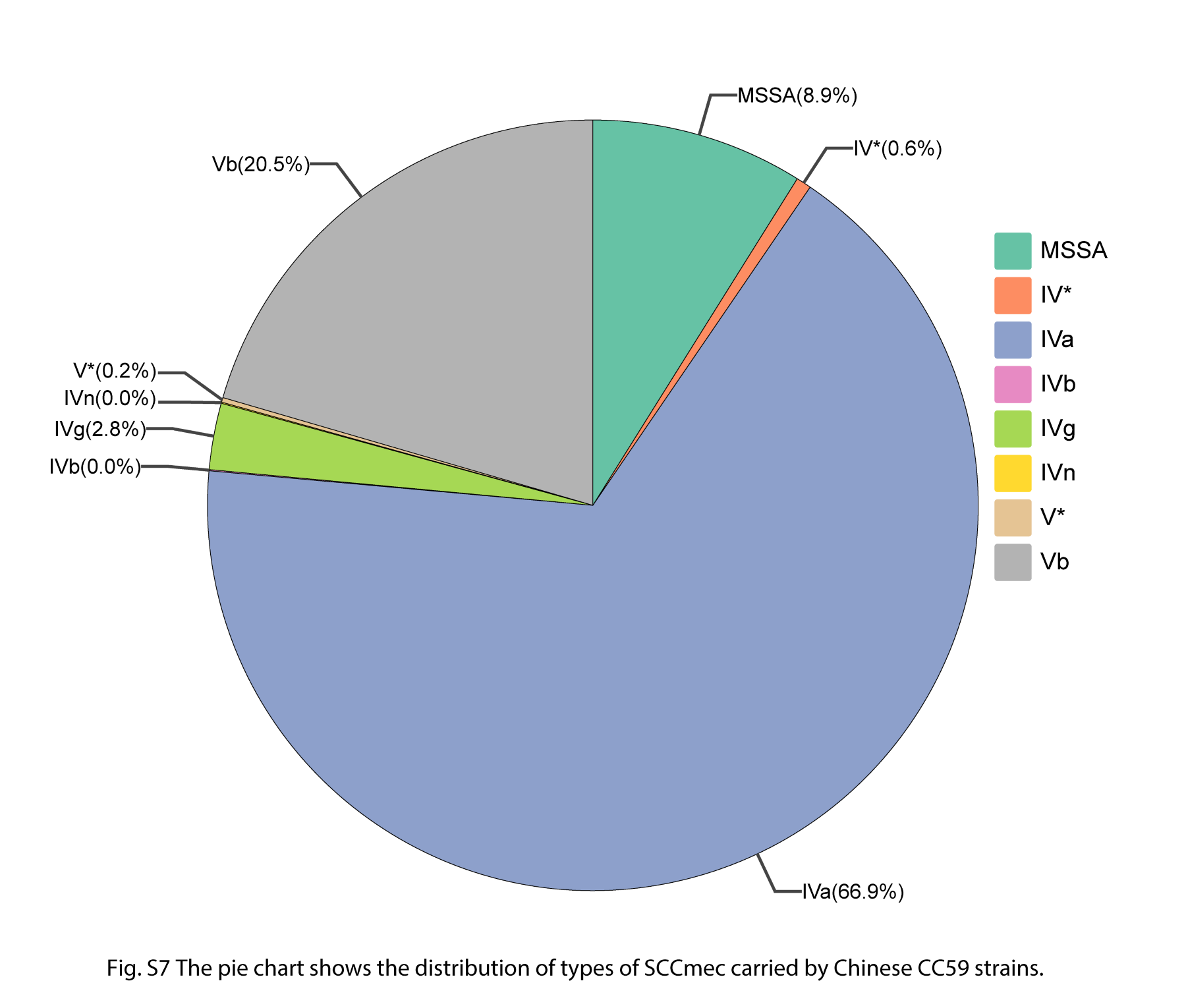

Supplement: Fig. S7 — Distribution of types of SCCmec carried by Chinese CC59 strains. [file msystems.01492-25-s0007.tif]

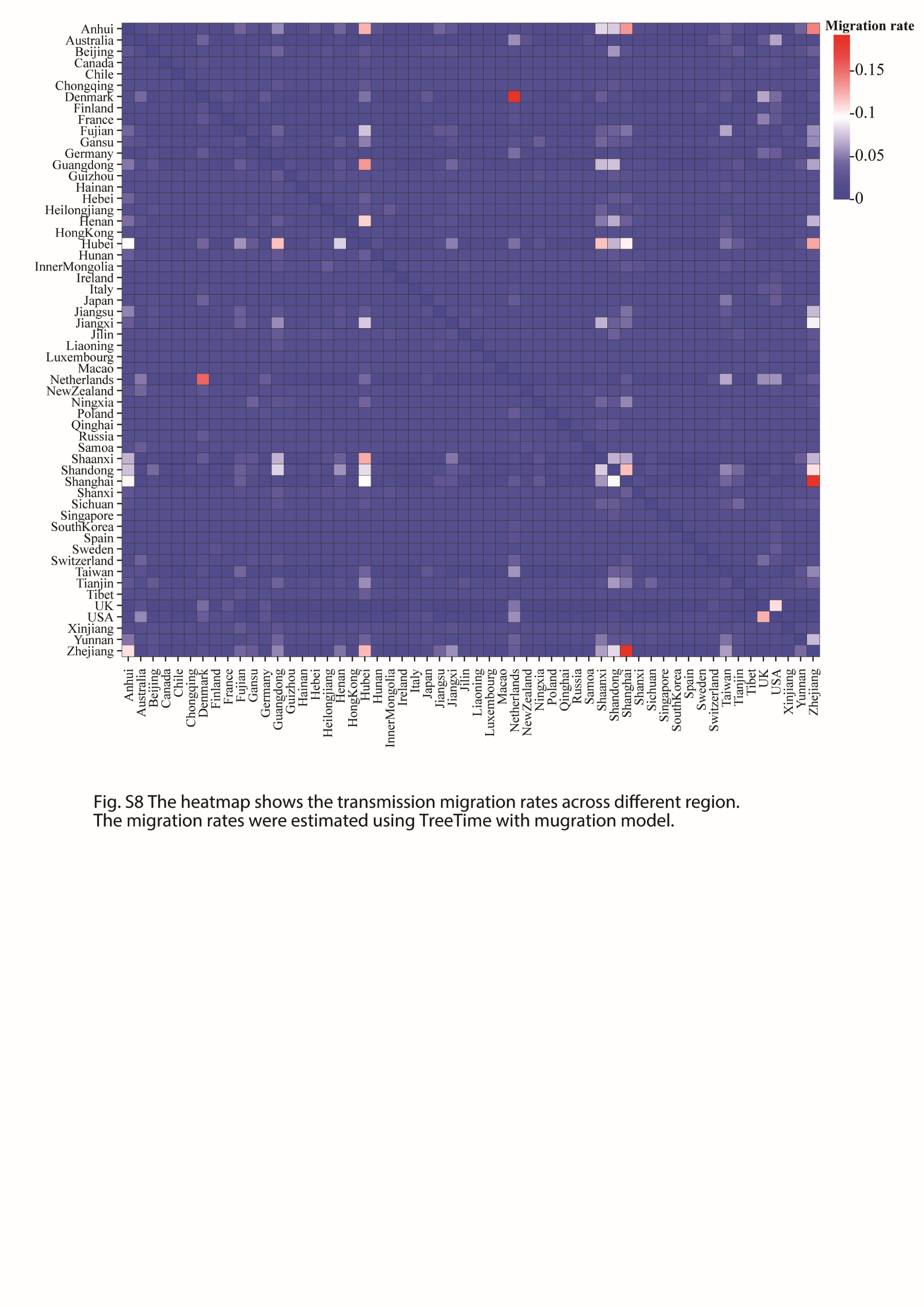

Supplement: Fig. S8 — Transmission migration rates across different region. [file msystems.01492-25-s0008.tif]
